# Impact of obesity on arterial health among young adults: A bioelectrical impedance study

**DOI:** 10.6026/973206300222985

**Published:** 2026-05-31

**Authors:** Dinakar Reddy D, Jagadamba A, Ashwini K. Shetty

**Affiliations:** 1 Department of Physiology, SDUMC, Sri Devaraj Urs Academy of Higher Education & Research, Tamaka, Kolar - 563101, Karnataka, India

**Keywords:** Obesity, arterial stiffness, pulse wave velocity (PWV), bioelectrical impedance, young adults

## Abstract

The relationship between obesity and early arterial stiffness in young adults remains inadequately understood despite the increasing prevalence of obesity-related cardiovascular risk. Therefore, it is of interest to investigate 132 healthy individuals aged 18-25 years to assess the relationship between obesity and arterial stiffness markers (PWV, Ao-PP, AIx) using bioelectrical impedance and oscillometric pulse wave analysis. Obese participants showed significantly higher Ao-PP and AIx (p < 0.05), while carotid-femoral PWV showed no significant difference. BMI had weak associations with arterial stiffness, whereas body fat percentage correlated significantly with Ao-PP and AIx. Thus, obesity in young adults is associated with early functional vascular changes, with body composition measures providing better early risk indicators than BMI.

## Background:

Cardiovascular disease (CVD) is the leading cause of global morbidity and mortality, with arterial stiffness recognised as an independent predictor of cardiovascular events and mortality [1]. Pulse wave velocity (PWV) and augmentation index (AIx), derived from non-invasive pulse wave analysis, are established markers of arterial stiffness and central hemodynamics [2]. Increased arterial stiffness reflects structural and functional arterial changes, resulting in reduced vascular compliance and impaired hemodynamics [3]. Obesity is a major modifiable risk factor for CVD, with excess adiposity contributing to vascular dysfunction through inflammation, endothelial dysfunction and autonomic imbalance [4]. Recent global evidence suggests that obesity prevalence has plateaued in several developed nations while continuing to rise rapidly in developing countries, increasing future cardiovascular risk burden among young populations [5]. Studies in adults show positive associations between obesity and arterial stiffness markers, including PWV and central pulse pressure [6]. In younger populations, findings are inconsistent, with some studies reporting increased arterial stiffness in overweight and obese individuals, while others show variability based on age and methodology [7, 8]. Measures of central adiposity may better predict vascular stiffness than BMI alone [8]. Central pulse pressure and AIx are considered early indicators of adiposity-related hemodynamic changes [10]. Therefore, it is of interest to investigate the associations between obesity and arterial stiffness, as well as central hemodynamic markers, in individuals aged 18-25 years.

## Methodology:

## Study design:

This cross-sectional observational study was conducted in the Department of Physiology at Sri Devaraj Urs Medical College, Kolar, following Institutional Ethics Committee approval and the Declaration of Helsinki.

## Sample size:

A total of 132 healthy individuals aged 18-25 years were included. Sample size was calculated using OpenEpi based on obesity prevalence in young adults, with 10% allowable error, 80% confidence level and 10% non-response adjustment.

## Study population:

Participants were recruited from the outpatient department using systematic random sampling, with every third eligible individual selected.

## Inclusion criteria:

Apparently healthy individuals aged 18-25 years who provided informed consent.

## Exclusion criteria:

History of cardiovascular disease, transient ischemic attack, varicose veins, pacemaker implantation, major systemic illness, or use of medications affecting cardiovascular function.

## Anthropometric assessment:

Height and weight were measured using standard methods and BMI was calculated (kg/m^2^). Obesity was defined using Asian- specific BMI cut-offs.

## Body composition analysis:

Body fat percentage was assessed using bioelectrical impedance analysis (TANITA) under standardized conditions. BIA is a validated, non-invasive method widely used in clinical and epidemiological studies [11,12]. However, variations between different bioimpedance technologies should be considered when interpreting body composition measurements, as complete agreement across devices may not always be present [13].

## Blood pressure measurement:

Brachial blood pressure was recorded using an automated oscillometric device after 5 minutes of rest. Three readings were taken at one-minute intervals and the mean was used. Pulse pressure was calculated as systolic minus diastolic pressure.

## Assessment of arterial stiffness:

Arterial stiffness and central hemodynamics were measured using an oscillometric ECG-gated pulse wave analysis system. Measurements were obtained in the supine position after 10 minutes of rest, following overnight fasting and avoidance of caffeine, smoking and physical activity. Parameters included cfPWV, baPWV, central aortic pressures, aortic pulse pressure, augmentation pressure, AIx and ABI. These methods are validated against tonometric techniques [14, 16].

## Grouping of participants:

Participants were categorized as obese (BMI ≥ 23 kg/m^2^ and elevated body fat percentage) or non-obese (BMI < 23 kg/m^2^ with normal body fat),improving identification of adiposity-related vascular risk beyond BMI alone [17].

## Statistical analysis:

Data were analysed using SPSS version 22.0. Normality was assessed using the Kolmogorov-Smirnov test. Variables were expressed as mean ± SD or median (IQR). Between-group comparisons were performed using independent t-test or Mann-Whitney U test. Spearman's correlation assessed associations and multiple linear regression evaluated obesity as an independent predictor. A p-value < 0.05 was considered statistically significant.

## Results and Discussion:

**Figure 1:**
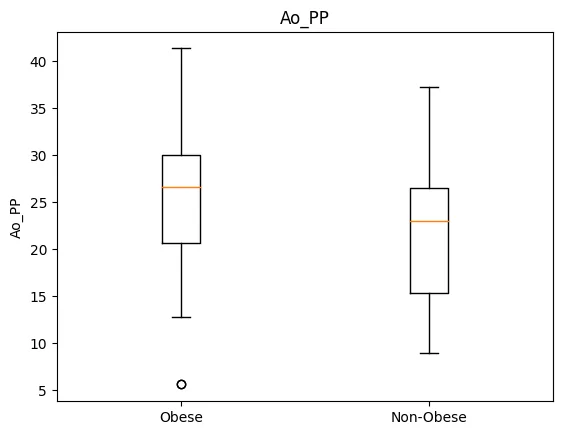
Comparison of aortic pulse pressure (Ao-PP) between obese and non-obese groups - Box-and-whisker plot showing distribution of aortic pulse pressure in obese and non-obese participants. The central line represents the median, the box indicates the interquartile range (IQR), and the whiskers denote minimum and maximum values. Aortic pulse pressure was significantly higher in obese participants (p = 0.003).

Participants were categorized into obese and non-obese groups based on Asian-specific BMI criteria. Arterial stiffness and central hemodynamic parameters were compared to assess the association of obesity with vascular function in young adults. Obese participants demonstrated significantly higher aortic pulse pressure (25.61 ± 7.18 mmHg vs 21.92 ±7.08 mmHg, p = 0.003) and aortic augmentation index (-5.14 ± 49.78% vs -13.20 ± 47.11%, p = 0.038) compared to non-obese individuals, indicating increased central hemodynamic load. However, carotid-femoral pulse wave velocity did not differ significantly between groups (691.17 ± 184.30 cm/s vs 727.23 ± 137.24 cm/s, p = 0.390) (Table 1). Correlation analysis showed weak, non-significant associations between BMI and arterial stiffness parameters, including aortic pulse pressure (r = 0.131, p = 0.281) and aortic augmentation index (r = 0.061, p = 0.615) (Table 2). Figure 1 illustrates the comparison of aortic pulse pressure between obese and non-obese groups, demonstrating significantly higher values in obese participants. Overall, obesity was associated with early alterations in central hemodynamic parameters without significant changes in carotid-femoral pulse wave velocity. This cross-sectional study demonstrates that obesity in young adults is associated with significant alterations in central hemodynamic parameters, even in the absence of detectable structural arterial stiffening. Obese participants showed significantly higher aortic pulse pressure and augmentation index compared to non-obese individuals, while carotid-femoral pulse wave velocity remained comparable between groups. These findings suggest that functional vascular changes may precede structural arterial alterations in early stages of obesity, consistent with previous observations in young populations [18, 19]. The observed increase in aortic pulse pressure among obese individuals indicates elevated central arterial load and reduced vascular compliance. Aortic pulse pressure reflects the pulsatile component of blood pressure and is influenced by arterial stiffness and wave reflection [15]. Increased values in obese participants may be attributed to early endothelial dysfunction, increased stroke volume and altered vascular tone associated with excess adiposity [3, 4]. Similarly, the higher (less negative) augmentation index observed in obese individuals reflects increased wave reflection and early changes in arterial function, as reported in obesity-related hemodynamic studies [9]. In contrast, carotid-femoral pulse wave velocity, considered the gold standard for assessing central arterial stiffness [14], did not differ significantly between groups. This suggests that structural stiffening of large arteries may not yet be established in this young population. The discrepancy between functional markers (Ao-PP and AIx) and structural markers (cfPWV) supports the concept that early vascular dysfunction precedes irreversible structural changes [20]. Correlation analysis revealed weak and non-significant associations between BMI and arterial stiffness parameters. This indicates that BMI alone may not adequately reflect adiposity-related vascular risk in young adults, as supported by studies highlighting the limitations of BMI in assessing body fat distribution [17]. In contrast, body composition measures such as body fat percentage provide a more accurate assessment of cardiovascular risk and show stronger associations with central hemodynamic parameters [10]. The mechanisms linking obesity to early vascular changes are multifactorial. Excess adiposity is associated with chronic low-grade inflammation, oxidative stress, endothelial dysfunction and increased sympathetic activity, all of which contribute to vascular dysfunction and arterial stiffening [6]. These pathophysiological processes lead to increased vascular tone and altered wave reflection, explaining the observed changes in aortic pulse pressure and augmentation index. Our findings are consistent with studies demonstrating increased arterial stiffness and central hemodynamic alterations in obese individuals across different age groups [21, 22]. However, studies in younger populations report variable results, likely due to differences in methodology and measurement techniques [7, 8]. The present study adds to existing evidence by showing that early hemodynamic changes are detectable even in apparently healthy young adults. The absence of significant differences in carotid-femoral PWV aligns with previous reports suggesting that measurable structural arterial stiffening occurs later in the disease process [2]. This highlights the importance of assessing sensitive early markers such as central pulse pressure and augmentation index for early cardiovascular risk detection. Clinically, these findings emphasize the importance of early identification of vascular alterations in obese individuals. Non-invasive assessment of central hemodynamic parameters may facilitate detection of subclinical cardiovascular risk before overt disease develops. Additionally, incorporation of body composition analysis may improve early risk stratification compared to reliance on BMI alone [11, 12]. The strengths of this study include the use of validated non-invasive techniques for arterial stiffness assessment and evaluation of multiple hemodynamic parameters in a young population. However, the cross-sectional design limits causal inference and longitudinal studies are required to establish temporal relationships and progression of vascular changes.

## Conclusion:

We show that obesity in young adults is associated with early functional alterations in vascular hemodynamics, preceding structural arterial changes. These findings underscore the clinical importance of early detection and highlight body fat percentage as a more sensitive marker than BMI for identifying subclinical cardiovascular risk and enabling timely preventive interventions.

## Advancement to knowledge:

This study demonstrates that obesity-related vascular changes may occur early in young adults and suggests that body fat percentage may be a more sensitive indicator of arterial dysfunction than BMI.

## Figures and Tables

**Table 1:** Baseline arterial stiffness and central hemodynamic parameters in obese and non-obese participants

**Parameter**	**Obese group**	**Non-obese group**
Carotid-femoral PWV (cm/s)	691.17 ± 184.30	727.23 ± 137.24
Aortic Pulse Pressure (mmHg)	25.61 ± 7.18	21.92 ± 7.08
Aortic Augmentation Index (%)	-5.14 ± 49.78	-13.20 ± 47.11
Values are expressed
as mean ± SD.
PWV = pulse wave velocity.

**Table 2:** Comparison of arterial stiffness parameters between obese and non-obese groups

**Parameter**	**Statistical test**	**p-value**
Carotid-femoral PWV	Mann-Whitney U	0.39
Aortic Pulse Pressure	Mann-Whitney U	0.003
Aortic Augmentation Index	Mann-Whitney U	0.038
P < 0.05 considered
statistically significant.
